# Secondary Forest Conversion Into Betel Nut Plantations Reduces Soil Water Retention by Altering Soil Properties

**DOI:** 10.1002/ece3.72924

**Published:** 2026-01-14

**Authors:** Ruiyu Fu, Qiaoyan Chen, Siyuan Cheng, Zhongyi Sun, Zhongmin Hu, Yangong Du, Licong Dai, Xiaowei Guo

**Affiliations:** ^1^ Key Laboratory of Adaptation and Evolution of Plateau Biota, Qinghai Haibei National Field Research Station of Alpine Grassland Ecosystem Northwest Institute of Plateau Biology Chinese Academy of Sciences Xining Qinghai China; ^2^ Hainan Baoting Tropical Rainforest Ecosystem Observation and Research Station, School of Ecology Hainan University Haikou China

**Keywords:** betel nut plantations, infiltration rate, secondary forest, soil physicochemical properties, soil water retention

## Abstract

Betel nut plantations have rapidly expanded in recent decades owing to their considerable economic benefits, resulting in a significant reduction of tropical secondary rainforests, which has had substantial impacts on soil hydrological properties. However, few studies have investigated the effects of forest conversion on these properties. In this study, soil samples from secondary forests (SF) and betel nut plantations (BP) were collected to assess soil physicochemical and hydrological properties. The results showed significantly higher topsoil (0–10 cm) water content and saturated water‐holding capacity in SF than in BP. However, the opposite pattern was observed in the subsurface soil layer (20–60 cm). Similarly, the 0–10 cm soil layer showed higher capillary water capacity and field capacity in SF than in BP, but this trend was reversed in the 10–60 cm soil layer. Additionally, the conversion of forests into betel nut plantations led to a reduction in saturated hydraulic conductivity. The soil hydrological properties were significantly affected by land‐use change through alterations in soil properties. We found that the soil nutrient content in BP was much lower than that in SF. Besides, soil capillary porosity played a role in influencing soil water retention, accounting for 32.03% of the variation, followed by total porosity (27.04%) and soil bulk density (26.5%), whereas the soil particle composition also had a resistance to soil degradation effect on soil water retention. Together, the conversion of secondary forest into betel nut plantations not only led to soil degradation, but also has negative effects on soil water retention capacity by reducing soil porosity and increasing soil bulk density. These findings provide important insights for the management of agroecological systems in tropical regions.

## Graphical abstract

Soil hydrological properties were significantly altered by the land‐use change. Topsoil water retention was reduced by the conversion of tropical forests into betel nut plantations. Stable soil infiltration rates were reduced by the conversion of tropical forests into betel nut plantations. Capillary porosity is the key control factor affecting soil water retention.

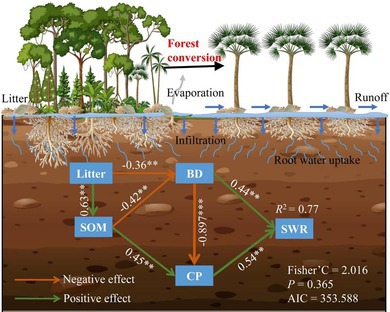

## 1 Introduction

Land‐use change, particularly the conversion of forests to agricultural plantations, is a predominant force shaping tropical landscapes and their ecosystem functions (Liu et al. 2025). Among the most rapid and extensive conversions in tropical Asia is the expansion of betel nut (*Areca catechu L*.) plantations. Driven by high economic returns and medicinal value, the global cultivated area of betel nut has experienced unprecedented growth, increasing from approximately 0.56 million hectares in 2000 to 1.24 million hectares in 2019 (FAO 2019). This expansion at the expense of primary and secondary tropical forests, leading to well‐documented declines in biodiversity (Ahrends et al. 2015), alterations in local microclimates (Liu, Liang, et al. 2019), and disruptions to nutrient cycling (Singh et al. 2021). Most importantly, the reduction in tropical rainforest area due to the embezzlement of betel nut plantations could significantly affect soil hydrological properties by altering soil and plant characteristics.

Soil ecosystem a fundamental component of forest functionality, undergoes significant degradation following conversion to intensive plantations (Feng et al. 2025). Numerous studies have demonstrated that betel nut cultivation negatively impacts soil quality relative to native forests. Several studies have consistently reported reductions in soil organic carbon stocks (Mishra et al. 2021; Kurmi et al. 2024), deterioration of soil physicochemical properties such as porosity and aggregate stability (Sun et al. 2021; Chen et al. 2019), and shifts in microbial community structure and function (Wang et al. 2017; Berkelmann et al. 2018). These changes are mechanistically linked to management practices associated with monocultures, including reduced litter input diversity, soil compaction from machinery or frequent human activity, and potential alterations in pH due to fertilizer application (Guillaume et al. 2015; Maranguit et al. 2017).

Soil water retention is a vital ecosystem property that governs a range of processes, including water availability for plant growth, groundwater recharge, and resistance to erosion and drought (Dai et al. 2023). This capacity is predominantly determined by soil physical characteristics, particularly porosity (which dictates water storage space) and organic matter content (which enhances water holding capacity by improving soil structure) (Yang et al. 2014; Dai et al. 2021). Given the negative effects of forest‐to‐plantation conversion on soil properties, such as reducing soil organic matter and compromising soil structure—it logically follows that soil water retention capacity would be adversely affected. Some indirect evidence supports this. For instance, several studies have reported increased surface runoff and soil erosion following the establishment of betel nut plantations, suggesting a reduction in the soil's ability to infiltrate and retain water (Cheng et al. 2008; Rahmat et al. 2018). However, a direct, mechanistic investigation into the specific soil property on soil water retention following forest conversion is still poorly understood.

Hainan Island accounts for 42.5% of the country's tropical land coverage (Li et al. 2022). In the past decades, betel nut plantations have been rapidly expanding in the lowland of tropical areas at the expense of reducing forested and agricultural lands (Sun et al. 2020; Zhai et al. 2014), leading to a significant reduction in tropical rainforest area. However, how tropical rainforest conversion to betel nut plantations affects soil water retention remains limited. To address these knowledge gaps, representative betel nut plantations and natural secondary forests were selected for comparative analysis, where both soil physicochemical and hydrological characteristics were assessed to examine how the conversion of tropical rainforests affects soil hydrology on Hainan Island. Specifically, the study aimed to explore how forest conversion affects soil hydrological properties and reveal their relationship with soil properties. We hypothesized that the conversion of secondary forests to betel nut plantations may not favor soil water retention due to soil physicochemical properties degradation. Our results may provide insights for forest conservation and management in the tropics.

## 2 Materials and Methods

### 2.1 Site Description

**FIGURE 1:**
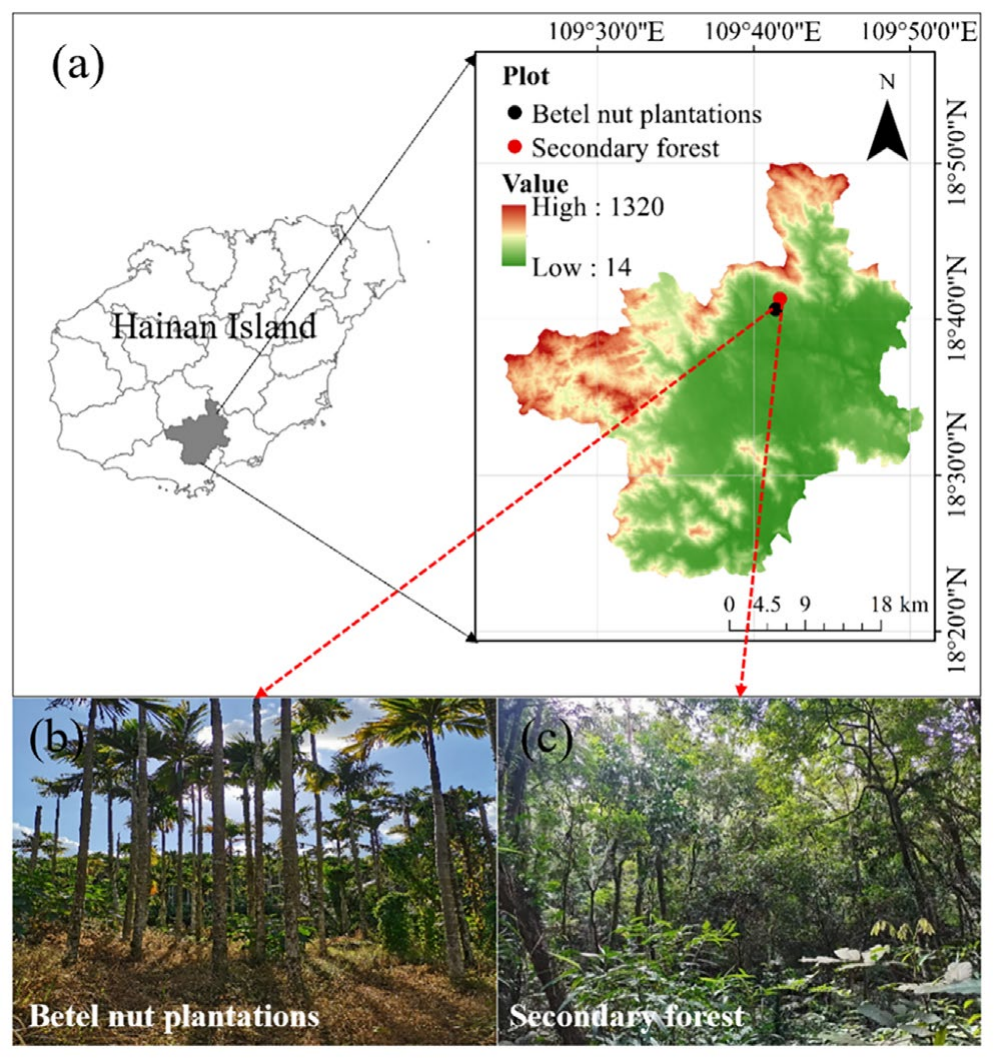
Study sites and two vegetation types (i.e., secondary forests and betel nut plantations).

Field studies were carried out in Baoting County (109°41′ E, 18°42′ N), located on Hainan Island, China (Figure 1). The region has a tropical monsoon climate with a mean annual temperature between 20.7°C and 24.5°C. The area receives annual precipitation of 1800–2300 mm, with clearly defined wet and dry seasons. The wet season (April–October) accounts for more than 80% of total rainfall, while the dry season persists from November to March (Chen et al. 2024). The USDA soil classification system identifies ferralsols and primitive soils as the primary soil types in this area (Sun et al. 2021). Economic incentives have led to the widespread conversion of Hainan Island's natural forests into betel nut plantations in the past decade, offering a valuable opportunity to investigate the impact of forest conversion (from secondary forests to betel nut plantations) on soil hydrological processes. Using historical logging data provided by regional forest management, along with increment borer samples analyzed in the laboratory, we obtained the age of secondary forests and betel nut plantations. Detailed information about the secondary forests and betel nut plantations is provided in Table 1. The dominant tree species in natural secondary forests are *Hopea hainanensis, Vatica mangachapoi, Vatica microcarpa*, *Wrightia pubescens, Sapindus saponaria, Mallotus anomalus, Ficus variegata Blume, Albizia chinensis*, and *Aglaia elaeagnoidea*. The dominant tree species in betel nut plantations is *Areca catechu* (betel nut).

**TABLE 1:** Basic information across vegetation types.

Vegetation types	Height (m)	DHB (cm)	Age (year)	Vegetation coverage (%)	Litter (t/hm^2^)
Secondary forest	11.16 ± 0.43a	22.9 ± 1.95a	25 ~ 30	80a	2.83a
Betel plantations	13.38 ± 0.21a	12.56 ± 1.40b	20 ~ 30	45b	1.68b

*Note:* Different lowercase letters above bars denote statistically significant variations (*p* < 0.05) between vegetation types.

Abbreviation: DHB, diameter at breast height.

### 2.2 Experimental Design and Soil Sampling

This study employed a space‐for‐time substitution (chronosequence) approach to infer the long‐term effects of converting secondary forests to betel nut plantations on soil hydrological functions. Field investigations were conducted in Baoting County, Hainan Island, China, in November 2022. Two distinct but comparable vegetation types were selected as endpoints of the assumed land‐use chronosequence: (1) secondary forests, representing the preconversion state, and (2) betel nut plantations, representing the post‐conversion state (Figure 1). It is critically important to note that all studied betel nut plantations were historically converted from secondary forest ecosystems based on detailed interviews with local landowners and examination of historical land‐use records. These plantations had been established for approximately 10 years and were abandoned at the time of sampling, with no recent fertilizer application. This abandonment ensured that any observed differences were more likely attributable to the legacy of the land‐use change itself, rather than to ongoing management practices.

To minimize the confounding effects of inherent environmental variation and strengthen the comparability between the two land‐use types, each betel nut plantation site was paired with a secondary forest site located within a spatial distance of less than 2 km; paired sites were selected to have closely matched altitude, slope gradient, and aspect, and the spatial proximity ensures that paired sites share similar geological background and soil parent material. For each vegetation type, three study sites (clusters) were established. Within each site, four replicate plots (20 × 20 m) were established. Five sub‐quadrat plots (1 × 1 m) were randomly set up within each large plot for soil sample collection. Soil samples were taken at six depth intervals (0–10, 10–20, 20–30, 30–40, 40–50, and 50–60 cm). Soils from the same depth across the five subquadrats were thoroughly mixed to form one composite sample per depth per large plot. A total of 72 composite soil samples were obtained (3 sites × 4 replicate plots × 6 depths) and transported to the laboratory for analysis of soil organic matter, particle size distribution, total nitrogen, total carbon, and the C: N ratio.

### 2.3 Laboratory Measurements and Analyses

To analyze the soil properties, the disturbed soil samples were sieved through 0.25‐mm and 2‐mm mesh sieves to eliminate roots and debris. Soil organic matter (SOM) was quantified following the Walkley & Black procedure (Nelson and Sommers 1982), while total carbon (TC) and total nitrogen (TN) were analyzed with an element analyzer (Elementar Vario EL III, Hanau, Germany). Particle size distribution (PSD) was measured using a Mastersizer 2000 (Malvern Instruments, UK). Bulk density (BD) was calculated as oven‐dry soil mass per 100 cm^3^ core volume. Furthermore, the soil total porosity (TP), capillary porosity (CP), and noncapillary porosity (NCP) were calculated using the equations provided by Dai et al. (2020).

(1) $$\mathrm{TP}=\left(1-\frac{\mathrm{BD}}{d_{s}}\right)\times 100\%$$

(2) CP=CWC×BD

(3) NCP=TP−CP

where TP, CP, and NCP denote the total porosity (%), capillary porosity (%), and noncapillary porosity (%) of the soil, respectively. CWC stands for the capillary water capacity, while ds refers to the soil particle density, which was estimated at 2.65 g/cm^3^.

### 2.4 Statistical Analysis

In our study, we adopted two‐way ANOVAs to investigate the impact of both vegetation types and soil depth, coupled with their interaction, on soil physicochemical and hydrological characteristics. To differentiate soil attributes between natural secondary forests and betel nut plantations, a one‐way ANOVA was conducted, followed by a post hoc least‐significant‐difference test at a 5% significance level for statistically significant divergences identified by ANOVA. In an effort to dissect the interplay between soil features and their hydrological effects, redundancy analysis (RDA) with the “vegan” package and partial Mantel testing methods were used. The R package of “rdacca.hp” was applied for a quantitative assessment and dissecting variance in our study (Lai et al. 2022). Besides, structural equation modeling (SEM) was utilized to determine the relationship between soil water retention and controlling factors. Statistical analyses were performed using R software (v4.0.3; R Core Team 2020). All results are presented as mean values ± SD.

## 3 Results

### 3.1 Variation in Soil Particle Size Distribution Across Two Vegetation Types

**FIGURE 2:**
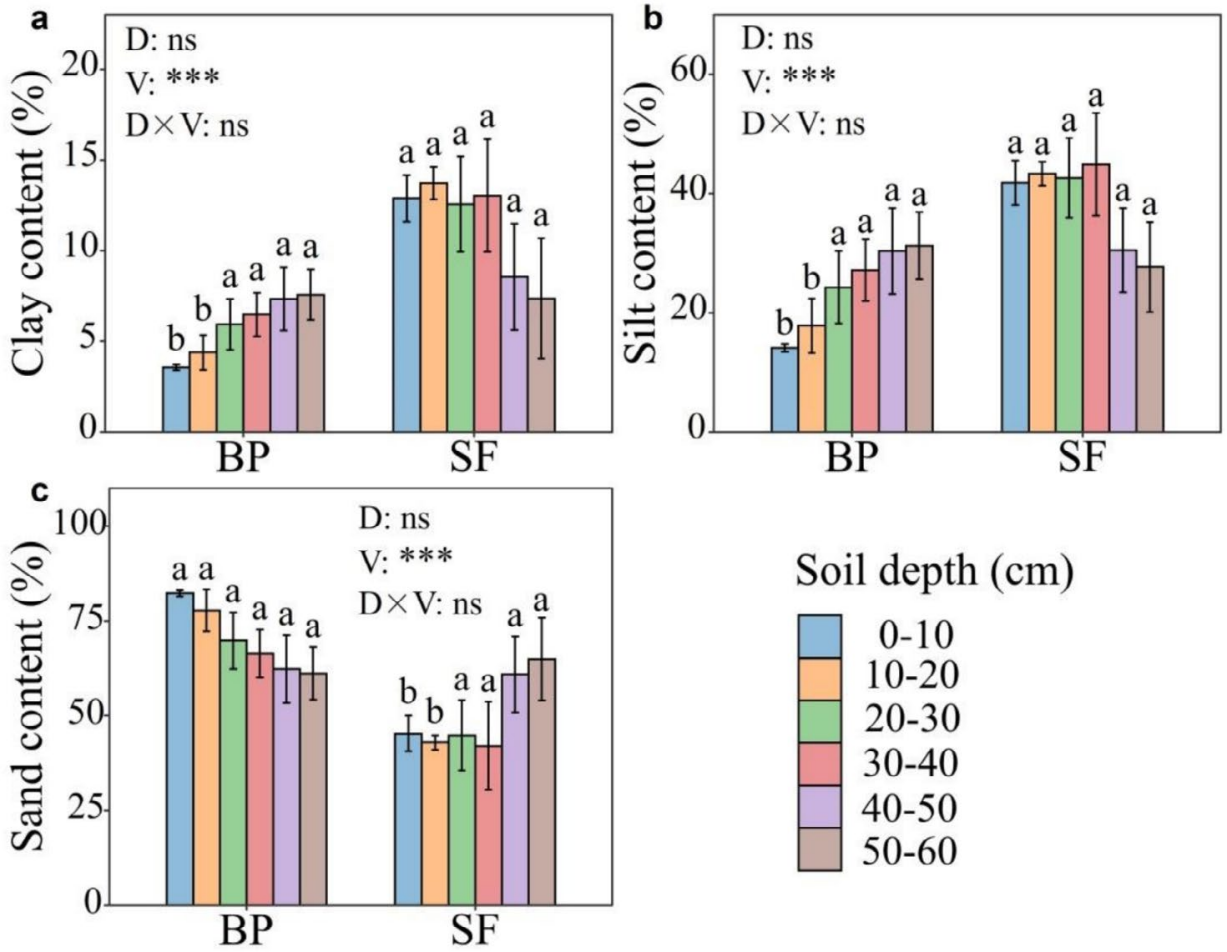
Soil particle size distribution across two vegetation types, (a) clay, (b) silt and sand (c). BP, betel nut plantations; SF, secondary forest. Different lowercase letters above bars denote statistically significant variations (*p* < 0.05) between vegetation types within each soil layer. V, vegetation type; D, soil depth; D × V, the interaction effect between soil depth and vegetation type. ***p < 0.001, ns denotes nonsignificant (*p* > 0.05).

The 0–60 cm soil particle composition was dominated by sand content (60% ± 3%) across the two vegetation types, followed by silt (mean 31% ± 2.25%) and clay (mean 9% ± 0.78%) (Figure 2). The soil particle size distribution was significantly different between betel nut plantations (BP) and secondary forest (SF) (Figure 2). Specifically, the 0–40 cm clay and silt content in SF was 1.95 and 1.45 times larger in the BP, with particularly significant differences (*p* < 0.001) observed in the 0–20 cm. In contrast, the 0–40 cm soil layer in SF contained 43.87% lower sand content than BP, especially for the 0–20 cm layer (*p* < 0.05). Besides, the clay and silt content in BP increased with soil depth, whereas the clay and silt content in SF displayed the opposite pattern. In comparison, the sand content reduced with soil depths in BP but displayed the opposite pattern for SF.

### 3.2 Variation in Soil Physicochemical Properties Across Two Vegetation Types

**FIGURE 3:**
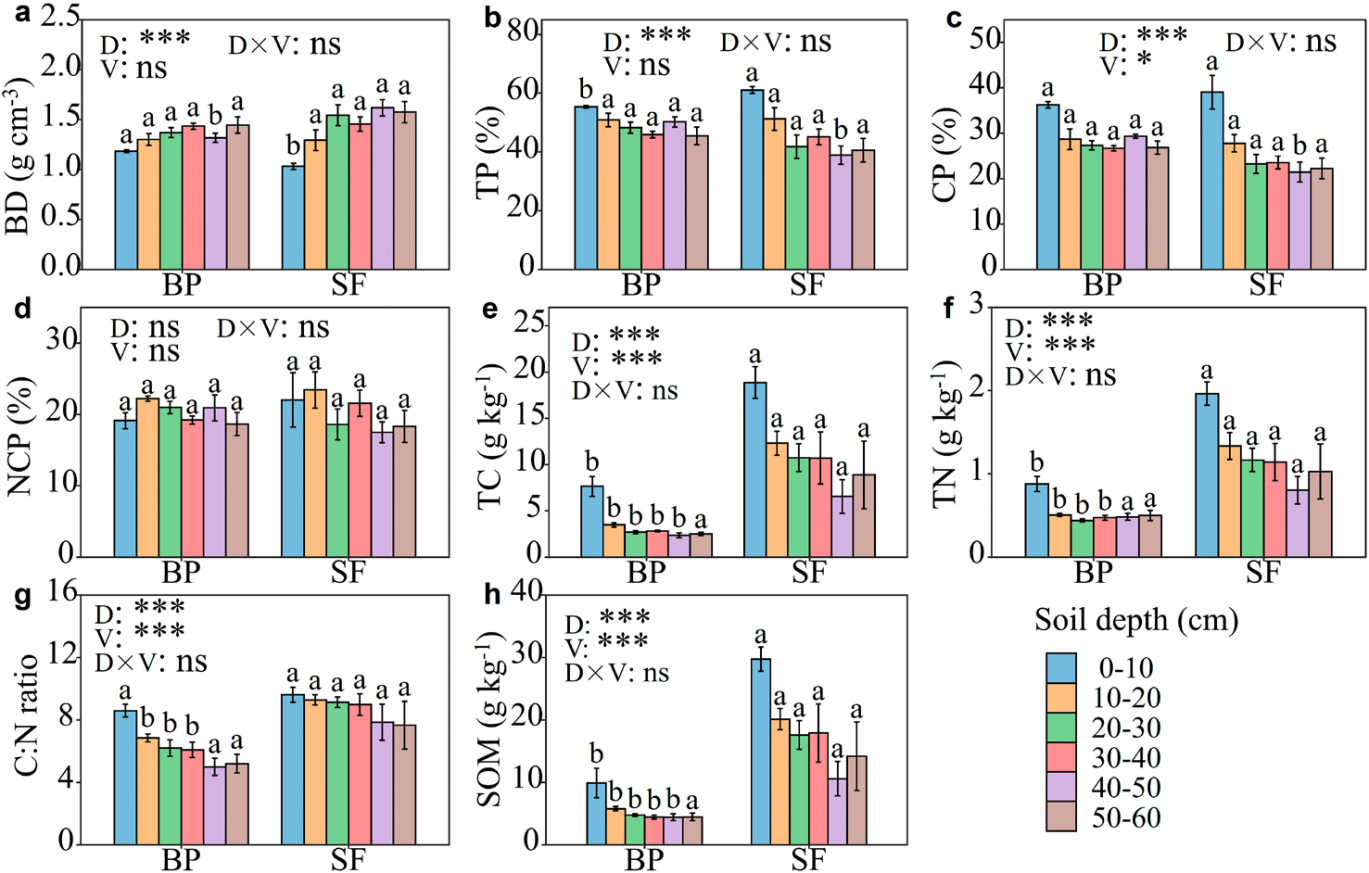
Soil physicochemical properties across two vegetation types. Distinct lowercase letters above bars denote statistically significant variations (*p* < 0.05) between vegetation types within each soil layer. BD (a), soil bulk density; TP (b), soil total porosity, CP (c), soil capillary porosity, NCP (d), soil noncapillary porosity; TC (e), soil total carbon; TN (f), soil total nitrogen, C: N (g), soil C: N ratio; CP; SOM (h), soil organic matter. D × V, the interaction effects between soil depth and vegetation type; D, soil depth; V, vegetation type. SF, secondary forests; BP, betel nut plantations. *p < 0.05, ***p < 0.001, ns denotes nonsignificant (*p* > 0.05).

Soil physicochemical properties were significantly affected by land‐use change. We found the 0–10 cm BD (1.03 g/cm^3^) in SF was significantly lower by 21% than that in BP (Figure 3a, *p* < 0.05), while TP and CP in SF were much higher than those in BP and reached statistical significance for TP (Figure 3b,c). In comparison, no significant variations were detected across the 10–60 cm, except for BD, TP, and CP at 40–50 cm. Additionally, the 0–40 cm SOM, TC, and TN in SF were 2.43, 2.16, and 1.43 times larger than in the BP (*p* < 0.001, Figure 3e–h). Similarly, the 0–10 cm C: N ratio in SF is 10% higher than that in BP, especially for the 10–40 cm (*p* < 0.05) (Figure 3g).

### 3.3 Variation in Soil Hydrological Properties Across Two Vegetation Types

**FIGURE 4:**
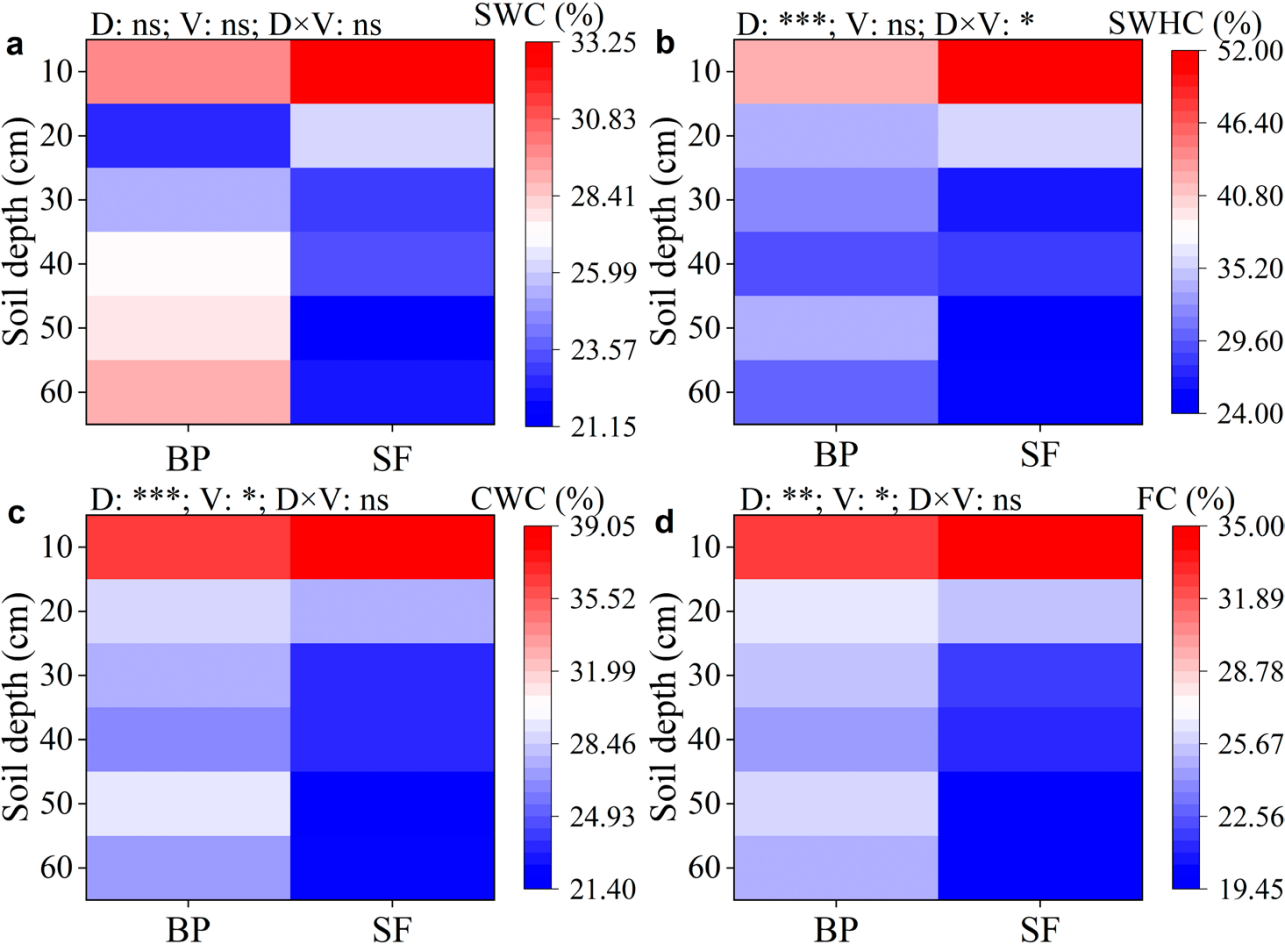
Soil water content (SWC) (a), saturation water‐holding capacity (SWHC) (b), capillary water content (CWC) (c), and field capacity (FC) (d) across two vegetation types. Different lowercase letters above bars denote statistically significant variations (*p* < 0.05) between vegetation types within each soil layer. BP, betel nut plantations; D × V, the interaction effect between soil depth and vegetation type; D, soil depth; BP, betel nut plantations; SF, secondary forest; V, vegetation type. *p < 0.05, **p < 0.01, ***p < 0.001, ns denotes nonsignificant (*p* > 0.05).

The soil hydrological properties differed significantly between BP and SF (Figure 4). We found the averaged SWC (27.03%), SWHC (33.53%), CWC (29.18%), and FC (26.53%) in BP across the soil profile were higher than those in SF of SWC (24.03%), SWHC (32.12%), CWC (26.04%), and FC (23.64%) (Figure 4). More specifically, topsoil (0–20 cm) SWC and SWHC in SF were 7% and 15% higher than those in BP (Figure 4a,b). In contrast, the deep soil (20–40 cm) water content and saturated water‐holding capacity in SF were 18.79% and 16.35% lower than those in BP (Figure 4a,b). Similarly, the 0–10 cm CWC and FC in SF were 7.74% and 7.56% higher than those in BP, whereas the 10–40 cm CWC and FC in SF were 14.86% and 15.35% lower than those in BP (Figure 4c,d). Two‐ANOVA results indicated significant effects of soil depth and depth×vegetation interaction on SWHC, whereas CWC and FC were significantly impacted by both soil depth and vegetation type separately (*p* < 0.05). Furthermore, as infiltration time increased, the infiltration rates decreased substantially, and SF had higher saturated hydraulic conductivity than BP (Figure S1).

### 3.4 Relationship Between Soil Physicochemical Properties and Hydrological Properties

**FIGURE 5:**
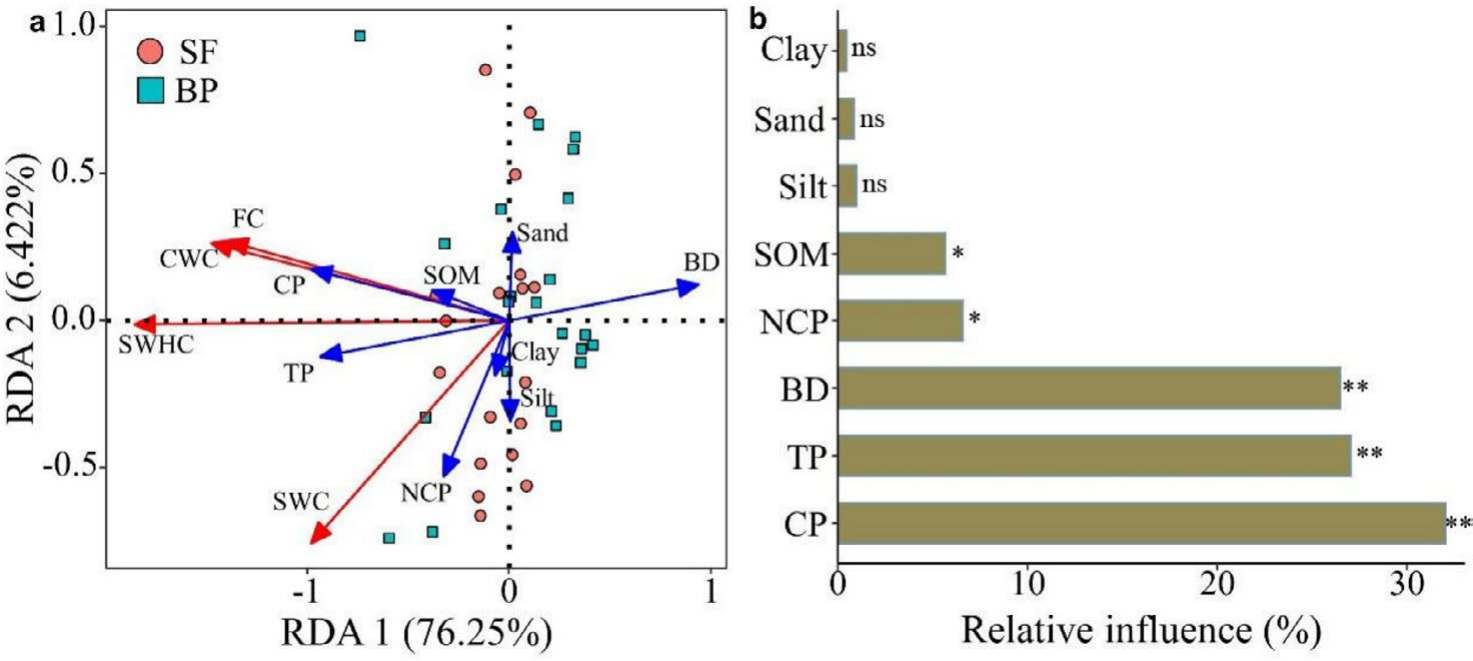
Redundancy analysis (a) and the relative influence (b) of soil properties on soil water retention. BP, betel nut plantations; SF, secondary forest. *p < 0.05, **p < 0.01, and ns denotes nonsignificant (*p* > 0.05).

**FIGURE 6:**
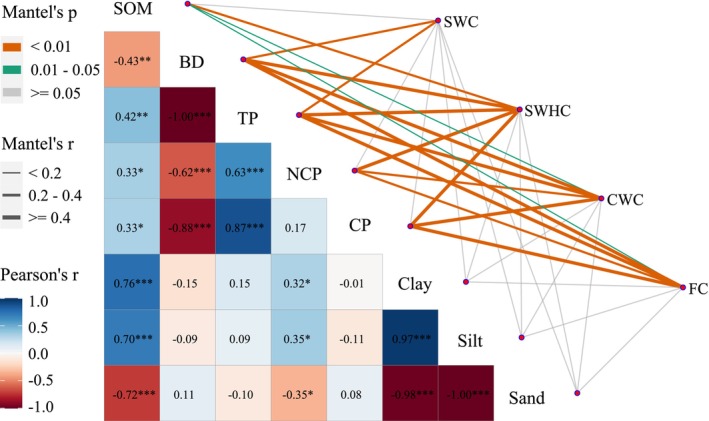
Partial Mantel tests between soil properties and soil water retention across two vegetation types. Color gradients represent the magnitude of spearman's correlation coefficients, line widths reflect Mantel's *r* values for distance correlations, and edge colors indicate statistical significance (*p* values) from 999 permutations.

**FIGURE 7:**
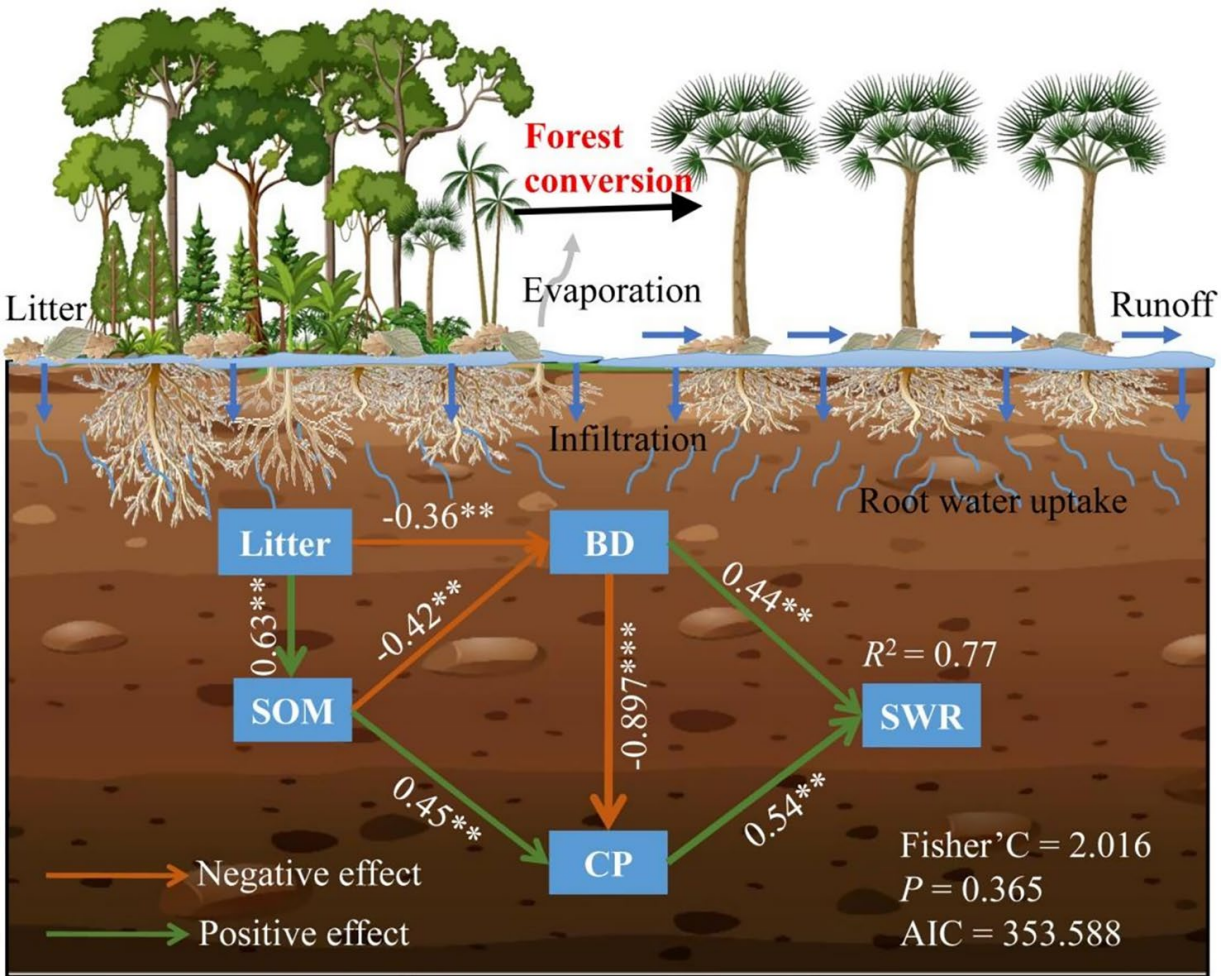
Schematic representation of structural equation model following secondary forest transformation into betel nut plantations. BD, topsoil bulk density; CP, capillary porosity; SOM, soil organic matter; SWR, soil water retention.

The RDA and partial Mantel test results consistently demonstrated that water retention capacity increased with SOM, TP, and CP but decreased with higher BD, clay, and silt contents. Soil texture showed weak effects on soil hydrological properties (Figures 5 and 6). The combined contribution of RDA1 and RDA2 explained 82.67% of the soil water retention variability (Figure 5a). Furthermore, the variance partition revealed CP as the dominant factor influencing soil water retention (32.03%), followed by TP (27.04%), BD (26.5%), NCP (6.57%), and SOM (5.66%). In contrast, soil particle size showed no significant influence on soil water retention (Figure 5b). The schematic representation shows modifications in soil characteristics and hydrological functions following the transformation from secondary forest to betel nut plantations (Figure 7).

## 4 Discussion

### 4.1 Effects of Land‐Use Change on Soil Hydrological Properties

The conversion of tropical forests to betel nut plantations significantly altered soil hydrological processes. Comparative analysis revealed greater topsoil water retention and saturated hydraulic conductivity in secondary forests relative to betel nut plantations (Figure 5), indicating weaker water conservation potential in betel nut plantations. These findings suggest that betel nut plantation topsoil exhibits greater susceptibility to both erosion and water stress. This aligns with prior research demonstrating that the conversion of tropical forests to betel nut plantations compromises soil water retention capacity (Cheng et al. 2008). Since soil water retention is primarily controlled by soil physicochemical properties (Chen et al. 2024; Dai et al. 2023). The contrasting retention capacities between vegetation types primarily contributed to variations in soil properties. Our results demonstrated that 82.67% of soil water retention variability could be accounted for by soil properties, confirming their dominant role in determining water retention. Specifically, we observed that soil organic matter exerts an indirect influence on soil water retention capacity via capillary porosity and bulk density (Figure 7). It was expected that the greater soil water retention in secondary forests might be attributed to their higher soil capillary porosity and lower bulk density compared to betel nut plantation. Consistent with previous research, we confirmed soil porosity as the dominant factor influencing soil water retention in tropical lowland ecosystems (Chen et al. 2025). Therefore, the greater soil water retention observed in secondary forests likely results from their enhanced soil porosity, especially capillary porosity. The effects of soil porosity on soil water retention are achieved by regulating the speed of water entering and moving through soils (Uteau et al. 2013). Soil capillary porosity profoundly influences soil water retention, shaping the intricate dynamics of water retention and movement within the soil matrix (Luo et al. 2022). This key soil characteristic is governed by capillary action, where the network of capillary pores allows water to be drawn upward from deeper soil layers or groundwater sources, ensuring continuous moisture availability for plants even during dry spells (Zhao et al. 2010).

In addition to soil properties, the litterfall facilitates the development of soil porosity, ultimately leading to a reduction in bulk density (Marschner et al. 2011; Pastore et al. 2020), which in turn promotes an increase in soil water retention. Compared to betel nut plantations, secondary forests display increased complexity in vegetation structure and composition, accompanied by higher litterfall inputs (Meli et al. 2024). This not only improves soil porosity but also mitigates erosion (Leite et al. 2018), potentially enhancing both soil water retention and infiltration. Meanwhile, considering that soil organic matter content is primarily derived from litterfall inputs, secondary forests may have higher soil organic matter owing to their higher litterfall levels, thereby improving soil pore structure and reducing bulk density, ultimately enhancing soil water storage capacity and stabilizing water availability. Additionally, our results revealed the topsoil layer water (0–20 cm) in secondary forests was higher than that of betel nut plantations, while the 20–60 cm SWC in betel nut plantations was higher than that in secondary forests (Figure 4a). The contrasting soil moisture and water retention capacity between natural secondary forests and areca nut plantations in 0–20 cm versus 20–60 cm layers arise from differences in transpiration, root distribution, and hydrological processes. Compared with areca nut plantations, secondary forests have deeper root distribution, combined with multilayered vegetation and high leaf area index (LAI), which have intense transpiration, thereby absorbing more water from deep soil layers, resulting in lower moisture content in the deep soil. In contrast, betel nut plantations (monocultures with lower LAI) have weaker transpiration, which consumes less water from deep soil, leading to a higher SWC. Besides, the roots of betel nut mainly concentrate in the shallow layer (0–20 cm) with minimal deep root development, leaving 20–60 cm water largely unconsumed (Sun et al. 2021).

### 4.2 Effects of Land‐Use Change on Soil Properties

Land‐use transformation substantially altered soil physical characteristics through both direct and indirect effects on biological components (e.g., litter deposition, species richness) (Li et al. 2019; Jiang et al. 2019). For instance, greater species abundance enhances root distributions and litterfall inputs, thereby improving soil structure (Zhu et al. 2019; Drewry 2006). Our results indicated a significant 21% decrease in surface soil bulk density for secondary forests compared to betel nut plantations, corresponding with enhanced capillary porosity and total soil porosity (Figure 3). These findings align with previous studies showing that tropical forests have higher soil porosity compared to rubber and tea plantations, especially in the topsoil layer (Li et al. 2012). Furthermore, we found that the silt and clay content was also higher in secondary forests compared to betel nut plantations. Our results suggested that transforming tropical rainforests into betel nut plantations does not improve soil structure. These discrepancies may be attributed to the community structure and species richness. Compared to betel nut plantations, secondary forests have more complex community structure and species richness, leading to higher litter quality and root systems. Previous studies found that soil bulk density was primarily affected by litter quality and root systems; higher litter quality and more extensive root systems can increase soil organic matter and promote soil aggregate formation, thereby contributing to a lower soil bulk density (Chen et al. 2024). Relative to secondary forests, the elevated bulk density observed in betel nut plantations, especially within the surface layer (0–10 cm), was probably caused by management practices like trampling and herbicide application, which likely promote compaction and correspondingly limit large pore formation.

As indicators of soil quality, soil chemical properties are also affected by land‐use change (Singh et al. 2020; Larreguy et al. 2017). In our study, the betel nut plantation influenced soil nutrients. Our study revealed substantially greater levels of topsoil nutrients (total carbon, total nitrogen, and soil organic matter) in secondary forests compared to betel nut plantations (Figure 4). These findings align with most previous studies demonstrating that natural forests contain greater quantities and reserves of soil organic matter, carbon, and nitrogen than monoculture systems (Tesfaye et al. 2016; Zhang et al. 2013), consequently enhancing soil quality relative to monocultures. These differences could potentially stem from variations in species abundance, understory vegetation, litter input, and root traits within natural systems (Xiong et al. 2008). Compared with secondary forest, betel nut plantations have much lower plant species abundance and understory vegetation, resulting in less litter production (Zeng et al. 2021). Given the organic matter input from plant litter and carbon mineralization rates, the higher input of litter in secondary forests results in higher soil nutrient content (Dai et al. 2023). Furthermore, the more diverse composition of secondary forests may produce higher‐quality litter than betel nut plantations, where monoculture conditions generate reduced litter quantities, aligning with established litter quality‐species richness relationships (Zhu et al. 2021; Schroth et al. 2002). The lower soil nutrients in betel nut plantations may also be associated with soil erosion owing to their lower soil water retention, especially considering that significant precipitation events occur frequently in the rain season. Besides, most betel nut plantations are established on slope, making the soil receive more rainfall drop kinetic energy and increasing the possibility of soil erosion, which eventually led to nutrient depletion and soil degradation (Zhu et al. 2018).

### 4.3 Implications for Ecosystem Services Management

In the past decade, the area of betel nut plantations has expanded rapidly in the tropical region due to its significant economic interests and important medicinal properties (Zhu et al. 2022). However, numerous studies have reported detrimental impacts on soil quality (Zeng et al. 2021; Widyati et al. 2022; Sun et al. 2021), including reduced carbon sequestration and soil fertility (Chen et al. 2017), accelerated soil acidification (Liu, Nie, et al. 2019), and altered soil microbial diversity (Monkai et al. 2018). Therefore, investigating changes in soil hydrological characteristics following the conversion of tropical rainforests to betel nut plantations is essential for maintaining ecological balance, particularly in highly productive tropical soils. In our study, we found that the conversion of tropical forest to a betel nut plantation has adverse effects on hydrological properties and soil nutrients. These findings align with existing studies reporting soil quality degradation after forest conversion to plantations (Sun et al. 2020, 2021). Land conversion reduces soil water infiltration and soil nutrient conditions; therefore, erosion prevention measures and nutrient supplementation should be implemented in betel nut plantations. More importantly, we found that soil porosity, especially capillary porosity, is the dominant driver of soil water retention, which is consistent with previous studies (Chen et al. 2024). Our results highlight the importance of considering ecosystem health and resilience in land‐use decisions. Meanwhile, the monoculture plantations typically have lower vegetation coverage and are more susceptible to resistant soil degradation, leading to the ecosystem becoming more vulnerable to biodiversity loss and climate change. Given the adverse effects on soil physicochemical and hydrological properties, caution should be exercised when the tropical rainforest is converted to betel nut plantations.

In addition to soil physicochemical and hydrological properties, land‐use change may also affect the socioeconomic conditions of the residents (Zheng et al. 2019). For instance, the conversion of natural forests into betel nut plantations has led to significant changes in the lifestyles of residents. Because the plantations are often located in remote areas, many residents have had to relocate closer to the plantations. This relocation not only changes their residential patterns but also affects the time allocation of their daily activities. For example, traditional farming activities are being replaced by plantation management, requiring residents to spend more time maintaining and managing these plantations. Besides, betel nut plantation enhances the economic income of the local residents at the cost of reducing the value of ecosystem regulation services. Therefore, there may be a trade‐off between pursuing economic benefit and maintaining ecosystem services. We recommend that intercropping an agroforestry ecosystem may be a practical solution for policymakers and practitioners to deal with the conflict. Previous studies have found that intercropping plantations significantly increase litter quantity and fine root biomass, thereby improving soil and water conservation relative to monoculture plantations (Wen et al. 2019). Therefore, future studies should be focused on the effects of complex ecosystem management in intercropped plantations on regulating services and promote regionally sustainable land use on Hainan Island.

## 5 Conclusion

We found that significantly greater water retention was in secondary forest topsoil relative to betel nut plantations, while deep soil layers showed opposite trends. Additionally, forest conversion to plantations substantially decreased saturated hydraulic conductivity. These combined effects of reduced topsoil water retention and infiltration capacity render plantation soils more susceptible to erosion and water stress. Moreover, our analysis revealed a strong correlation between soil water retention capacity and changes in soil properties; the soil structure was deteriorated by increasing soil bulk density and reducing soil clay and silt content; and soil nutrients were reduced following the land‐use change. Soil water retention capacity was mainly influenced by soil porosity, especially capillary porosity. Thus, the reduced water retention observed in betel nut plantations likely stems from their decreased soil porosity. Our results suggested that conversion tropical forests into betel nut plantations has adverse effects on both soil physicochemical properties and hydrological properties. Therefore, in the context of the global conversion of forests to plantations, there may be a tradeoff between pursuing economic benefit and maintaining ecosystem services. We recommend that intercropping an agroforestry ecosystem may be a practical solution for policymakers and practitioners to deal with the conflict. Our results could provide implications for sustainable monoculture plantations management.

## Author Contributions

**Ruiyu Fu:** conceptualization (equal), data curation (equal), investigation (equal), writing – original draft (equal). **Qiaoyan Chen:** data curation (equal), investigation (equal), visualization (equal). **Siyuan Cheng:** data curation (equal), investigation (equal), resources (equal). **Zhongyi Sun:** conceptualization (equal), methodology (equal), writing – review and editing (equal). **Yangong Du:** conceptualization (equal), methodology (equal), writing – review and editing (equal). **Zhongmin Hu:** conceptualization (equal), methodology (equal), writing – review and editing (equal). **Licong Dai:** conceptualization (equal), methodology (equal), writing – review and editing (equal). **Xiaowei Guo:** conceptualization (equal), methodology (equal), writing – review and editing (equal).

## Funding

This work was supported by the Collaborative Innovation Center for ecological civilization from Hainan University (XTCX2022STB07, XTCX2022STC01), CAS “Light of West China” Program (grant No. xbzglzb2022031), Independent Initiated Project from Environment and Plant Protection Institute, Chinese Academy of Tropical Agriculture Sciences (hzs2024003), Hainan Provincial Natural Science Foundation of China (422QN264), start‐up funding from Hainan University (KYQD(ZR)‐22085), Hainan Province South China Sea New Star Science and Technology Innovation Talent Platform Project (NHXXRCXM202303), and National Natural Science Foundation of China (42207524, 42361144877, U23A2002).

## Conflicts of Interest

The authors declare no conflicts of interest.

## Supporting information


**Figure S1:** saturated hydraulic conductivity across two vegetation types. BP, betel nut plantations; SF, secondary forest.

**Data S1:** ece372924‐sup‐0002‐DataS1.xlsx.

## Data Availability

The data for this manuscript is available as supporting information—data.
