# Supplementary material for: Secondary Forest Conversion Into Betel Nut Plantations Reduces Soil Water Retention by Altering Soil Properties
Source: Ecol Evol. 2026 Jan 14;16(1):e72924. doi: 10.1002/ece3.72924 (PMC12800915; doi:10.1002/ece3.72924)
Supplement: Supplementary file 1 — Figure S1: saturated hydraulic conductivity across two vegetation types. BP, betel nut plantations; SF, secondary forest. [file ECE3-16-e72924-s002.docx]

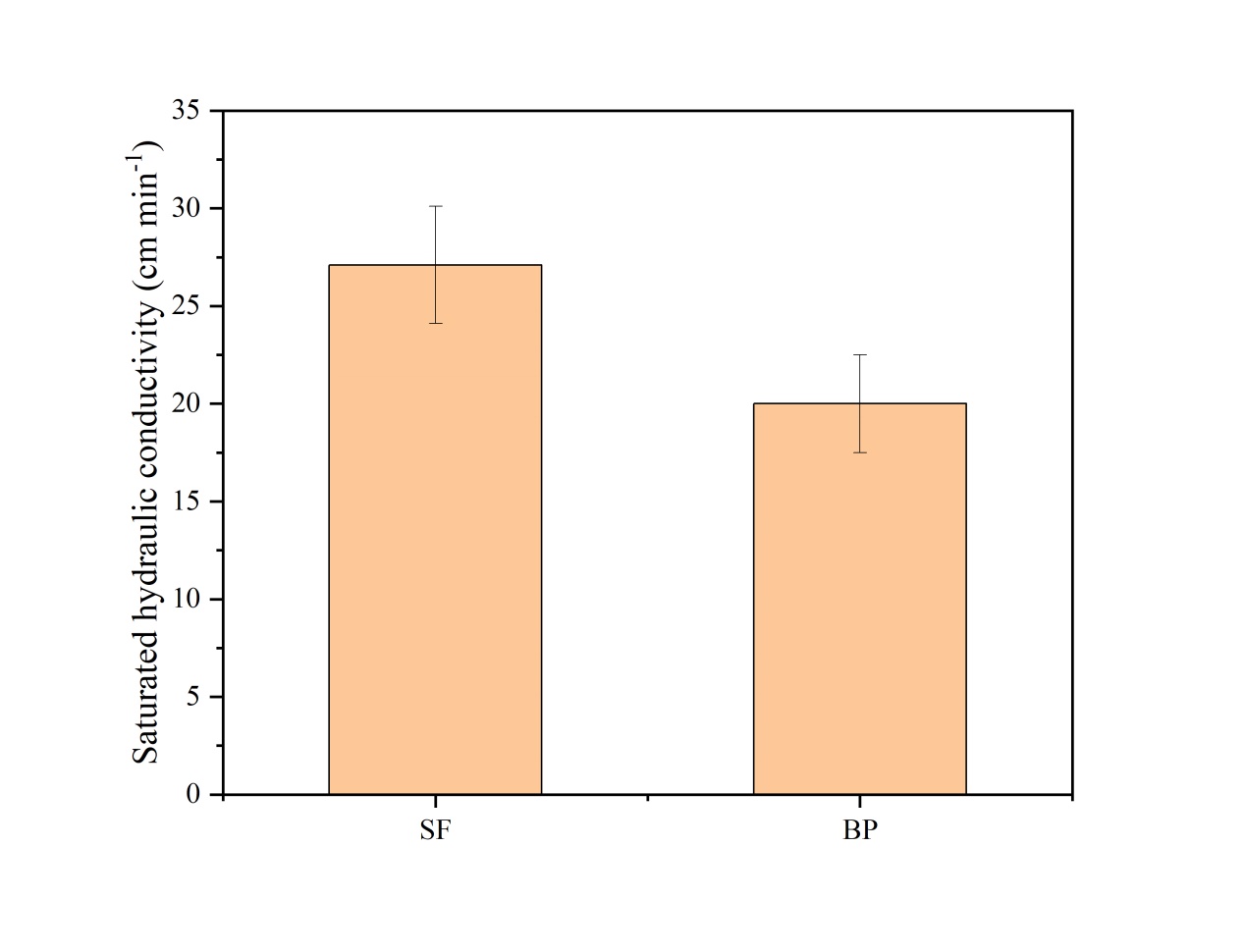


Fig. S1 saturated hydraulic conductivity across two vegetation types. Note: BP, betel nut plantations; SF: secondary forest.
